# Clinical Lectures on Diseases of the Nervous System

**Published:** 1898-05

**Authors:** 


CLINICAL LECTURES ON DISEASES OF THE NERVOUS SYSTEM. Delivered at the National Hospital for the Paralyzed and Epileptic, London. By W. R. Gowens, M. D., F. R. S., Physician to the Hospital; Consulting Physician to the University College Hospital; formerly Professor of Clinical Medicine in University College. Published by P. Blakiston, Son & Co., Philadelphia. 1012 Walnut Street. 1895. Price, Cloth, $2.

The work consists of twenty clinical lectures, on various conditions of nervous disease. The author is one of the most distinguished English authorities on this subject, and his work is always valuable because original. The work is a good companion piece to the one recently written by S. Weir Mitchell in this country, and strangely, though both works purport to be lectures on clinical cases taken at random, they do not trench upon each other in any way, as they do not in any instance discuss the same subject. Thus the books supplement each other and are equally valuable.

Chapters especially interesting are those on Mistaken Diagnosis, The Fort Clonus and its Meaning, Neuralgia and Optic Neuritis. The work is somewhat marred, though not materially injured, by its involved syntax, and errors in proof reading.

